# Design of a Bio-Inspired Untethered Soft Octopodal Robot Driven by Magnetic Field

**DOI:** 10.3390/biomimetics8030269

**Published:** 2023-06-22

**Authors:** Ruomeng Xu, Qingsong Xu

**Affiliations:** Department of Electromechanical Engineering, Faculty of Science and Technology, University of Macau, Avenida da Universidade, Taipa, Macau 999078, China; yc27952@um.edu.mo

**Keywords:** soft robots, bio-inspired design, magnetic actuation, medical robots

## Abstract

Inspired by insects in nature, an increasing number of soft robots have been proposed to mimic their locomotion patterns. As a wireless actuation method, the magnetic actuation technique has been widely applied to drive soft magnetic robots for diverse applications. Although recent works on soft materials have stimulated the development of soft robots, it is challenging to achieve the efficient movement of soft robots for in vivo biomedical application. Inspired by centipede locomotion, a soft octopodal robot is designed in this paper. The robot is fabricated by mixing magnetic particles with silicone polymers, which is then magnetized by a specific magnetic field. The prototypes can be actuated by an external magnetic field (5–8 mT) produced by custom-made electromagnetic coils. Experimental results show that the soft robot can move at a high speed in the range of 0.536–1.604 mm/s on different surfaces, including paper, wood, and PMMA. This indicates that the soft robot can achieve comparable speeds to other robots, while being driven by a lower magnitude, resulting in energy savings. Furthermore, it achieves a high speed of 0.823 mm/s on the surface of a pig colon. The fine capabilities of the soft robot in terms of crossing uneven biological surfaces and carrying external loads are demonstrated. The results indicate that the reported soft robot exhibits promising applications in the biomedical field.

## 1. Introduction

Soft robots are gaining more and more attention because of their high flexibility, large number of degrees of freedom (DOF), and easy fabrication. Such merits meet more requirements which cannot be fulfilled by traditional rigid-body robots [1,2,3,4]. Soft robots are inspired by different living creatures [5], such as the inchworm’s crawling and steering [6], millipede’s crossing complex terrains [7], octopus’s autonomous navigation [8], turtle’s adaptive morphogenesis in multiple environments [9], and fish’s fast mobility [10]. The flexibility of soft-bodied animals in nature allows them to change the shape and behavior of their bodies to adapt to a variety of complex environments [11,12,13]. In the meantime, due to the advancement of materials science, small-sized robots have been developed in recent years, which are expected to enable a wide range of medical applications, including targeted drug delivery, minimally invasive surgery, and cell manipulation [14,15,16,17].

Currently, ongoing research focuses on micro-robots capable of performing intricate tasks in both terrestrial and liquid environments. These robots are driven by a variety of actuation strategies, including physical, chemical, and biological methods for propulsion [18]. However, chemical propulsion faces challenges in the precise control of chemical reactions and potential harm to organisms [19]. Similarly, the development and fabrication of bio-driven robots are accompanied by significant complexities [20]. Consequently, researchers have shown considerable interest in physical propulsion mechanisms. In the pursuit of medical applications, the utilization of driving methods such as electric actuation and piezoelectric actuation is restricted due to the inherent limitations imposed by control circuitry. Notably, optical and magnetic actuation methods offer the potential for wireless propulsion. However, optical actuation brings inherent risks associated with tissue damage, while magnetic actuation has demonstrated biocompatibility [21]. As a result, compared to other actuation strategies, magnetic actuation is considered one of the most promising technologies for biomedical applications, because of its good properties, such as untethered control, biocompatibility, rapid response, harmless human–machine interaction, and low cost [22,23,24]. Small-scale robots can be driven by a magnetic field. The induced magnetic force or magnetic torque is produced by combining an external magnetic actuation system and a small magnet material inside the robot body [16,25,26].

With the increasing interest in magnetic soft robots, researchers have explored various fabrication methods to meet the demands of this field. In recent years, 3D-printing technologies, including extrusion-based 3D printing [26,27], light-based 3D printing [28], and UV photolithography [29], have gained great attention [14]. These techniques offer the advantage of achieving intricate structural designs. However, their implementation often requires expensive experimental equipment, limiting their accessibility for many researchers. In contrast, traditional methods such as molding and casting present cost-effective alternatives for fabricating structures that meet the desired performance requirements. These methods have been widely employed in various fields due to their simplicity and affordability. However, regarding film structures, spin coating and scratch coating prove to be more suitable options [7]. These techniques allow for the deposition of thin films with precise control over thickness and uniformity. Their versatility in accommodating different substrates makes them favorable choices for film-based applications. On the other hand, their limitations in achieving thicker and more rigid structures make them less suitable for other types of composites. In this study, we adopted the molding approach as our fabrication method for constructing magnetic polymer composites (MPCs) [30]. By incorporating the magnets of various sizes, ranging from micro-/nanometer to millimeter scale, into soft materials, we aimed to harness the benefits of both magnetic functionality and mechanical flexibility. The molding approach offers the advantage of versatility, enabling the production of complex shapes and customized designs. Furthermore, it facilitates the integration of magnetic particles into the polymer matrix, ensuring a homogeneous distribution and enhancing the overall performance of the resulted MPCs. Through the utilization of the molding approach, we sought to contribute to the field of magnetic soft robots by exploring the potential of incorporating magnets into the polymer matrix to achieve desired functionalities and improved performance.

Generally, two main factors govern the performance of these MPCs. One is that the type and morphology of magnetic particles and their volume fraction affect the magnetic strength. The other is that the polymer matrix determines their mechanical properties [31]. The type and morphology of the magnetic particles, along with their volume fraction, play a crucial role in determining the magnetic strength of the composites. Different magnetic materials exhibit varying levels of residual magnetization and internal magnetic field stability once magnetized. NdFeB (neodymium iron boron) is a widely recognized example of a hard magnetic material known for its high residual magnetization and stable internal magnetic fields [32]. On the other hand, the choice of the polymer matrix significantly impacts the mechanical properties of the MPCs. Various polymer options are available, including viscous liquids with Newtonian-like properties, hydrogels, and elastomers [33]. Among these choices, silicone rubber stands out as the most commonly used polymer matrix in MPCs due to its favorable attributes. Silicone rubber offers ease of handling during the fabrication process and possesses the ability to cure rapidly at room temperature, making it convenient for practical applications. In summary, the combination of suitable magnetic particles, such as NdFeB, with a compatible polymer matrix, such as silicone rubber, enables the development of high-performance MPCs with enhanced magnetic strength and desirable mechanical properties. The understanding of the roles of magnetic particles and polymer matrices is crucial for optimizing the design and fabrication of magnetic soft robots and other related devices.

In the literature, many studies have demonstrated that soft robots can be propelled by magnetic force in liquids. To name a few, Diller et al. developed a millimeter-scale swimming robot that can be magnetically driven in the liquid with a low Reynolds number, which is similar to an in vivo environment [34]. Ren et al. designed a sheet robot that can achieve multi-modal motion, such as rolling, wave crawling, wave swimming, and spiral crawling [35]. However, these robots possess a simple membrane structure, which makes it difficult for them to maintain high-speed motion by carrying an external load. It has been shown that multi-legged robots exhibit good load-carrying capacity and can move at higher speeds. For example, Venkiteswaran et al. designed a variety of multi-legged robots capable of navigating various terrains, including sharp triangular peaks and smooth sinusoidal surface [36,37]. Another notable example is a multi-legged soft millirobot developed by Lu et al., achieving an impressive locomotion speed of up to 40 limb lengths per second. This robot exhibits the ability to traverse simulated wet and dry gastric environments [7]. These robots have been effectively validated for overcoming various types of obstacles and navigating simulated human environments. Nevertheless, the application of magnetic soft robots in actual biological environments poses great challenges, particularly when it aims at achieving high locomotion speeds under low magnetic magnitudes. This challenge arises due to the presence of a mucus layer that often adheres to the surface of biological tissues, resulting in considerable surface friction force. The increased friction force on the surface requires a higher driving force during the initiation of motion, which typically translates to higher magnetic field strength and lower mass requirements. In recent years, Wu et al. developed a wireless-control soft millirobot capable of maneuvering through porcine tissues [38]. However, a notable limitation of this robot is its slow locomotion speed.

**Figure 1:**
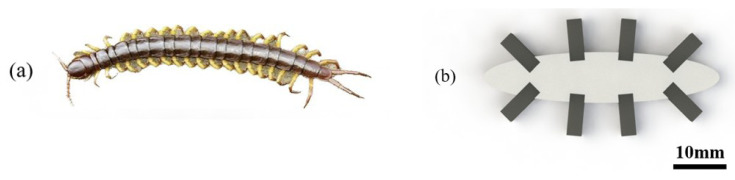
A magnetic soft robot inspired by the reptile, centipede. (**a**) Photo of a centipede with multiple feet in nature. (**b**) The soft robot inspired by the centipede.

In nature, many multi-legged animals employ a walking pattern known as alternate bipedal contacting with the ground, including humans, ants, and centipedes. Intriguingly, despite being a multi-legged creature, centipedes exhibit an alternating gait pattern where each pair of legs makes contact with the ground. This walking mechanism enables higher locomotion speeds and enhanced obstacle traversal capabilities. In small-sized organisms such as insects, the majority of them exhibit either hexapodal or multi-legged locomotion, while quadrupedal locomotion is relatively uncommon. It is hypothesized that quadrupedal structures exhibit lower stability compared to other leg configurations. For instance, the larval stage of ladybugs is quadrupedal, but they possess elongated tails to enhance their overall structural stability and prevent overturning during climbing. Similarly, compared to hexapodal locomotion, multi-legged locomotion provides greater stability. Therefore, it is conjectured that multi-legged robots possess stronger obstacle traversal capabilities. By constructing and testing quadrupedal and hexapodal robots, and considering comprehensive factors, such as robot size suitable for intraluminal navigation, we propose the design, fabrication, and actuation of a novel centipede-inspired octopodal robot in this paper, as depicted in Figure 1. The feet of the robot are made of MPCs magnetized under a specific magnetic field (with a certain direction), and the body part is made of a silicone rubber matrix (non-magnetic part). Compared to other magnetic particles, NdFeB particles are more feasible to produce magnetic torque because they are magnetically isotropic hard magnetic particles. After magnetization, the soft robots can move in a completely untethered manner driven by external electromagnetic coils.

The main contribution of this work is the development of an untethered soft-body octopodal robot to overcome existing challenges of traditional robots toward biomedical application. Experimental investigations were carried out to demonstrate its capability of moving at an average speed of 0.823 mm/s on the surface of a pig colon along with a certain load-carrying capacity. The remaining parts of the paper are organized as follows. The design and fabrication processes of the soft robot are presented in Section 2.1. Section 2.2 dictates the actuation method of the magnetic soft robot. Experimental results and discussion are reported in Section 3. Section 4 concludes this work.

## 2. Materials and Methods

Insects in nature have become the inspiration for soft-bodied robots due to their simple structure and environmental adaptability. To mimic the extremely adaptable properties, a bio-inspired soft octopodal robot is designed in this section.

### 2.1. Mechanism Design

In this study, we conduct further investigation into the correlation between leg length and foot-to-foot spacing in various terrestrial animals, with the aim of determining an optimal ratio for the design of a bio-inspired soft octopodal robot. Our observations indicate that longer legs have the potential to enhance the robot’s overall locomotion speed by enabling larger strides and increased velocity. However, it is crucial to consider the trade-off between speed and support. While longer legs promote faster motion, they would degrade the robot’s stability and its ability to provide adequate support. On the other hand, a larger foot-to-foot distance enhances support and stability but reduces the robot’s locomotion speed. These findings are consistent with the broader patterns observed in terrestrial animals. Through our extensive review, we identified a prevalent leg length to foot-to-foot spacing ratio ranging from 1 to 2 among the majority of legged animals. This range reflects the natural adaptation of animals to balance speed and support in their locomotion. Building upon this understanding, we obtained inspiration from the structure of centipedes and designed the leg length to foot-to-foot spacing ratio as 1:1 for the soft octopodal robot. This specific ratio yields a balance between efficient locomotion and adequate support. By maintaining an equal proportion between leg length and foot-to-foot spacing, the robot’s legs possess the necessary flexibility for optimal locomotion efficiency, while simultaneously ensuring sufficient support for the body during the movement.

The structures of conventional soft robots, with each part constructed by magnetic particles, are simple and easy to fabricate. However, these simple structures limit the diversity of robot deformations. Some parts of the robot containing magnetic materials (which are magnetized in different directions) can be designed into different shapes and sizes. By connecting the non-magnetic soft parts in different ways, a variety of soft robot structures can be obtained to achieve multi-functionality. Although many soft robots have been reported for various application fields, it is still difficult for them to move in real in vivo environments at high speed.

**Figure 2:**
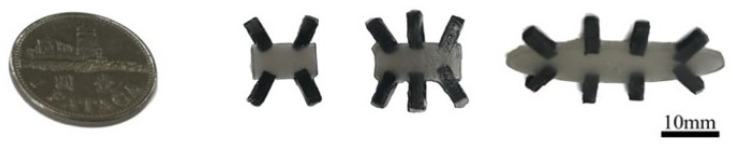
Prototype of quadrupedal, hexapodal, octopodal robots, and a 1-pataca coin.

**Figure 3:**
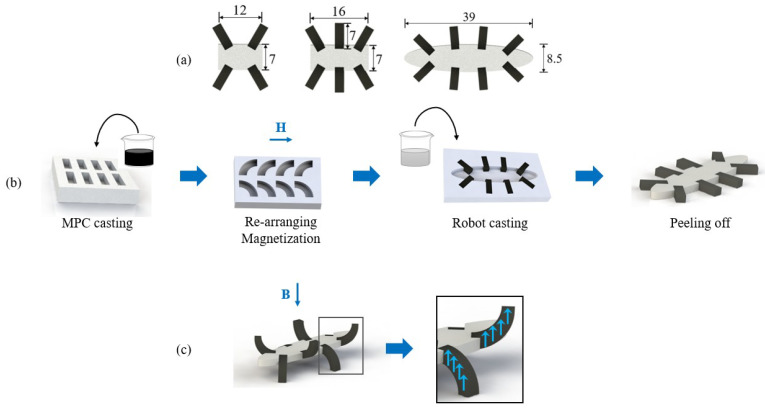
Design and fabrication process of the soft robots. (**a**) Dimensions of robots are in millimeter scale as shown in the top view. (**b**) Schematic of the robot fabrication process, exemplified by the octopodal robot. Mixture containing NdFeB powder and silicone rubber is firstly poured into a mold for curing. The cubes are rearranged and magnetized in the magnetic dipole direction (blue arrow). After casting the silicone rubber in the mold, the robot is peeled off from the mold. (**c**) Schematic of deformation of the robot, exemplified by the octopodal robot.

Inspired by polypod animals in nature, an octopodal robot is designed to reduce the friction force between the robot and harsh contact surface to increase the locomotion speed. In this work, the feet of the robot are magnetized at curved shapes, which can be transformed into curved shapes under an external magnetic field. The adjacent feet bend in different directions, which allows the robot’s body to be lifted from the floor. Because its contact area with the floor becomes smaller, the friction force also becomes smaller. It indicates that the robot possesses a strong ability to cross barriers thanks to the smaller contact area. In the meantime, the robots are expected to move at a high speed in water because there is no large friction. We also designed quadrupedal and hexapodal robots for comparison, using the same leg structure. Figure 2 shows the fabricated prototypes of the designed soft robots. The main dimensions of the robots are illustrated in Figure 3a.

### 2.2. Fabrication Method

Figure 3b illustrates the fabrication procedure of the soft robots, exemplified by the octopodal robot. Similar fabrication procedures are applied to other robots. In this work, the adopted magnetic material is NdFeB powder. The polymer is a silicone rubber (Ecoflex-0010), which was used for fabricating the non-magnetic part of the soft robot. Because of its low modulus of elasticity (55.2 kPa at 100% strain) and high elongation at break (>700%), it is well suitable as a soft part of the robot. We also considered the relationship between the proportion of NdFeB powder in the mixture and the required magnetic field intensity for robot actuation. It was observed that a higher proportion of NdFeB powder leads to a decreased magnetic field intensity requirement for robot movement. However, when the ratio of NdFeB powder to silicone rubber reaches 3:1, adhesion between adjacent legs occurs, which hinders the smooth movement of the robot. To address this issue and to ensure both an adequate magnetic force and unrestricted movement, we adopted a ratio of 2:1 for the NdFeB powder to silicone rubber.

The polymer is first obtained by mixing a mixture of two liquid precursors at 1:1 mass ratio. Next, the NdFeB powder is mixed into it at a mass ratio of 2:1. After being degassed in a high vacuum chamber for 3 min to remove air bubbles, the polymer liquid is filled into the mold (which is made by 3D printing) and cured at room temperature for 4 h to cast the MPC. Subsequently, the cured MPC (legs of the soft robot) are re-arranged in a specific shape to obtain the desired magnetization direction by supplying a 1.8 T magnetic field for 2 s. Afterward, the silicone rubber matrix (i.e., the mixture mentioned above) is poured into another mold with the MPC arranged. It is cured under the same conditions as above. Finally, the soft robot is peeled off from the mold.

By controlling the direction of the external magnetic field, the soft robot gains a specific deformation due to the magnetization profile as shown in Figure 3c. It is observed that four legs can lift the soft robot off the floor, while the other four legs stay in the opposite direction to decrease the area between the robot and the floor. This facilitates the locomotion of the soft robot on the floor surface.

### 2.3. Magnetic Actuation Method of the Soft Robot

The magnetic moments of the hard-magnetic materials are all aligned in the same direction as the external magnetic fields. In the meantime, magnetic materials gain high remanent magnetization. After magnetization, they can be actuated by the force ($F_{m}\in R^{3}$) and torque ($T_{m}\in R^{3}$), which are applied to the magnetic dipoles. They are expressed as follows:

(1) $$F_{m}=▽\left(m\cdot B\right)={\left[\frac{\partial B}{\partial x}\frac{\partial B}{\partial y}\frac{\partial B}{\partial z}\right]}^{T}m$$

(2) $$T_{m}=m\times B$$

where m ($m\in R^{3}$) is the magnetic moment of the soft robot and B is the strength vector of the external magnetic field. The magnetic force ($F_{m}$) and torque ($T_{m}$) deform the polymer due to the mechanical stress of the magnetic materials. Thus, the deformation of the soft robot can be predicted by controlling the magnitude and direction of the external magnetic field.

**Figure 4:**
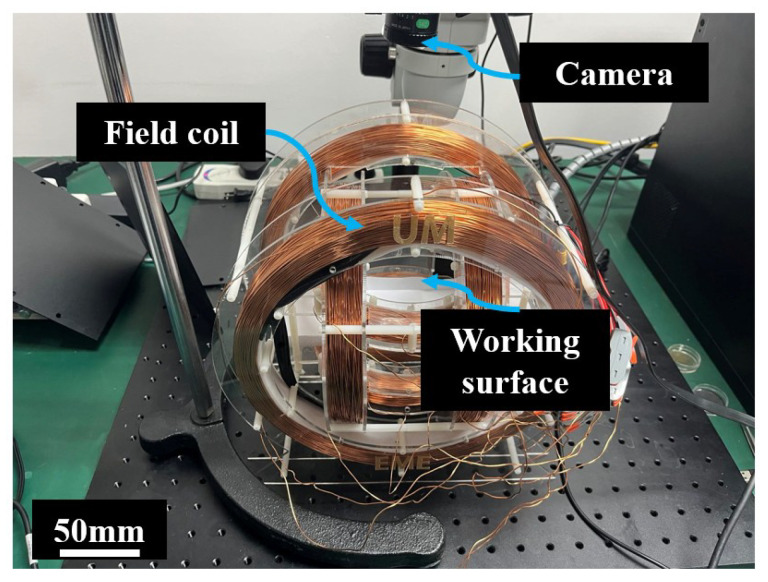
Prototype and experimental setup of the magnetic actuation system.

In this work, the robots are actuated by external electromagnetic coils. The coils are assembled around a workspace (50×100 mm${}^{2}$) as shown in Figure 4. A camera is adopted to observe and record the locomotion of the soft robots.

The octopodal robot has two groups of legs with different directions of magnetization. Due to the magnetization of the robot, the magnetic particles are actuated by the magnetic torque. The dynamic locomotion of the soft robot is regulated by a rotating magnetic field (B). The magnetic field is produced in the X-Z plane rotating around the *Y* axis, where the *Z* axis is vertically upward, and the *X* axis is along the direction of the robot locomotion.

For producing the locomotion, the magnetic field is shown as follows:

(3) ω=2πf

(4) θ=ωt

(5) $$B_{z}=B\sin\omega t$$

(6) $$B_{x}=B\cos\omega t$$

where *f* is the frequency of the rotating magnetic field.

**Figure 5:**
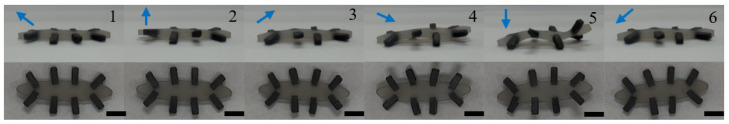
Images of the robot locomotion testing. The blue arrows depict the directions of the external magnetic field. Scale bar, 10 mm.

Figure 5 shows the snapshots of the robot locomotion in one cycle. Owing to the magnetization directions of the legs, the rotating magnetic field in the X-Z plane produces a straight-line locomotion. The legs are lifted in an alternating manner. The change of direction is easily achieved by controlling the field.

## 3. Results and Discussion

In this section, experimental studies are conducted to demonstrate the locomotion capability of the fabricated soft robot. The magnetic actuation techniques for the movement of the robot and their influence on the magnetization profile are tested. The locomotion performance is characterized. Finally, the testing results in harsh environments demonstrate the versatility of the proposed soft robot.

### 3.1. Results of Robot Locomotion Testing

**Figure 6:**
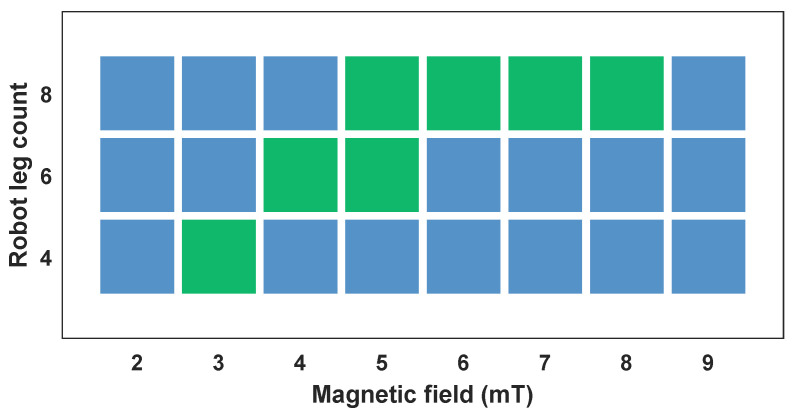
Initiation of quadrupedal, hexapodal, and octopodal robots under different magnetic field magnitudes on a paper. Blue squares indicate failure or unstable robot motion, while green ones indicates successful locomotion.

To verify the hypothesis as mentioned earlier, an experimental comparison study was conducted among the three types of robots, and the results are shown in Figure 6.

The results indicate that the number of legs on a robot influences the performance of magnetic propulsion. When the magnitude is relatively low, it is insufficient to drive the robot. The results reveal that robots with fewer legs require a smaller minimum magnitude for activation. This is because the quadrupedal robot has the least mass, resulting in a lower force requirement for propulsion. However, as the magnetic field magnitude increases, the quadrupedal robot is the first to tip over due to its poor stability. This poor stability can be attributed to two factors. First, the relatively small mass of the robot results in a reduced gravitational force acting on it. Second, when the magnitude is high, the legs that bend upward experience an increased magnetic force. Because the quadruped robot has a relatively short body length, it is unable to balance the torque generated by the magnetic field, leading to rolling over.

From the testing results, it is observed that although the octopodal robot requires a relatively large magnitude of magnetic field for activation, it exhibits a broader range of stable and controllable magnetic fields B, ranging from 5 to 8 mT. This finding confirms our initial hypothesis. Simultaneously, it can be demonstrated that a larger driving magnetic field is not necessarily better. Hence, a suitable magnetic field is needed for practical application.

**Figure 7:**
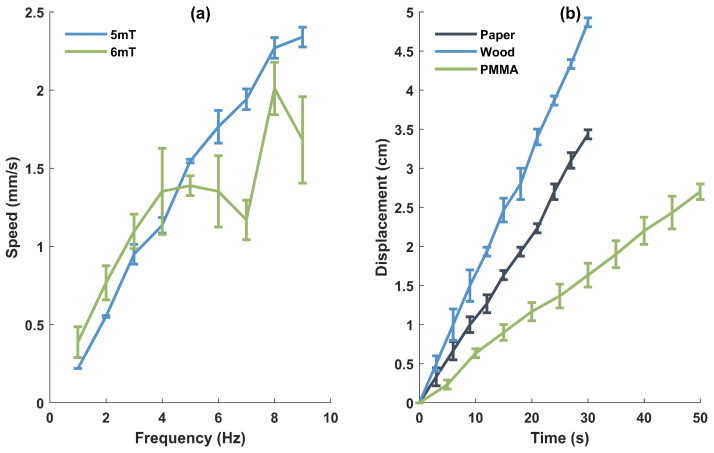
(**a**) Motion speed of the octopodal robot under different frequencies and magnitudes of the magnetic field on paper. (**b**) The motion displacements of the octopodal robot on three types of substrate surfaces.

Moreover, separate tests were conducted to test the motion speeds of the octopodal robot on a paper at the magnitudes B of 5 mT and 6 mT, under various frequencies. The results are presented in Figure 7a.

The results demonstrate that increasing the frequency of the magnetic field enhances the speed of the robot movement. However, when the magnitude becomes larger, raising the frequency does not significantly contribute to improving the robot’s motion speed. This is primarily due to a decrease in the stability of the robot as the magnetic field strength increases as discussed earlier. This also indicates that there is a certain limit to increasing the speed by raising the frequency.

The octopodal robot can move at a high speed on the surfaces of different materials. In this study, unless otherwise specified, for achieving stable robot movement and facilitating observation, the testing conditions are set as B = 5 mT and *f* = 4 Hz.

Figure 7b shows the displacements on different surface materials, where the mean errors are depicted. The efficacy of the motion can be compared in terms of locomotion speed. Table 1 tabulates the speed on each material in two forms, i.e., displacement per second and body length per second. The results show that the robot moves at the fastest speed on the wood board, reaching up to 1.604 mm/s. In contrast, it exhibits the slowest speed on PMMA surface at 0.536 mm/s due to the larger friction force between the robot and surface.

The discrepancy can be attributed to the variations in frictional coefficients between the robot and different surface materials. The higher speed on the wood board can be attributed to its higher friction coefficient, which results in a larger frictional force. This increased frictional force enables better traction for the robot, allowing it to generate more propulsive force against the surface. As a result, the robot can traverse the surface more quickly. On the other hand, the slower speed on the PMMA surface is primarily due to its lower friction coefficient, resulting in a smaller frictional force. This reduced frictional force leads to less effective traction and reduced propulsive force, thus hindering the robot’s movement. By analyzing the robot’s performance on different surface materials, we can observe the direct influence of frictional coefficients on locomotion speed. These findings emphasize the importance of considering the properties of the contact surface when designing and operating soft robots to optimize their performance in various environments.

To assess the impact of the magnetic actuation method on the robot’s locomotion speed, we conduct a comparison study versus similar robots in the literature as presented in Table 2. In this study, we compare the maximum achievable speeds of robots across different research articles, considering the differences in surface materials utilized. The first three robots in the table exhibit relatively high locomotion speeds, particularly the Millipede robot, which achieves a remarkable speed of up to 1.68 B.L. per second. The driving mechanisms employed by these robots are different, which accounts for their high speeds. All of the three robots utilize the approach of moving permanent magnets to propel the robot, which means that the robot’s movement speed is largely determined by the speed of motion of the magnets. Additionally, the use of permanent magnets allows the generation of stronger gradient magnetic fields, resulting in faster robot movement. By comparing the subsequent robots’ speeds, it can be observed that the octopodal robot, compared to the robots of the same type, can be operated under a lower magnitude of B = 5 mT while achieving a faster locomotion speed of 0.040 B.L. per second.

### 3.2. Results of Robot Function Testing

The versatile function of the octopodal robot is demonstrated by the locomotion testing in harsh environments below, including heavy loading, a stomach model in a dry/wet environment, and a surface of a pig colon.

**Figure 8:**
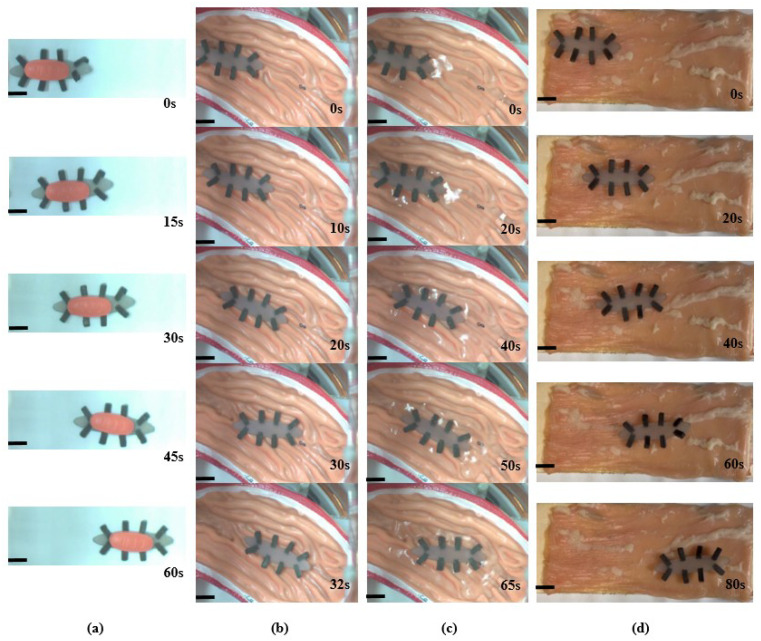
Experimental results. (**a**) Snapshots of the octopodal robot transportation test with an external loading. (**b**) Images of the octopodal robot locomotion in a stomach model under the dry environment. (**c**) Images of the octopodal robot locomotion in a stomach model under the wet environment. (**d**) The locomotion of the octopodal robot on the surface of a pig colon. Scale bar, 10 mm.

Under the external field, the robot can move at 0.695 mm/s on a paper by carrying a drug of 1.717 g, as shown in Figure 8a. The fact that the drug stays on the robot during the transporting proves that the locomotion is feasible. For the purpose of observation, the experimental parameters are set as **B** = 9 mT and *f* = 5 Hz.

To explore more potential biomedical applications, the locomotion test of the soft robot in a human stomach model is conducted as shown in Figure 8b,c. To obtain a stronger driving magnetic field, we adopted the approach of rotating a permanent magnet. The internal structure of the stomach is complex with 1.3–4.2 mm in depth and 1.2–5.4 mm in width. The robot moves faster in a dry environment as shown in the left part and slower in water environment as shown in the right part in Figure 8c. The slowest speed occurs at the junction. There are two main factors that contribute to this effect. First, the friction on the ground and the resistance in the water are different. Second, the grooves in each part of the model are different. By controlling the external magnetic field, the soft robot is navigated to the target location, which shows that the robot is highly manipulable.

**Figure 9:**
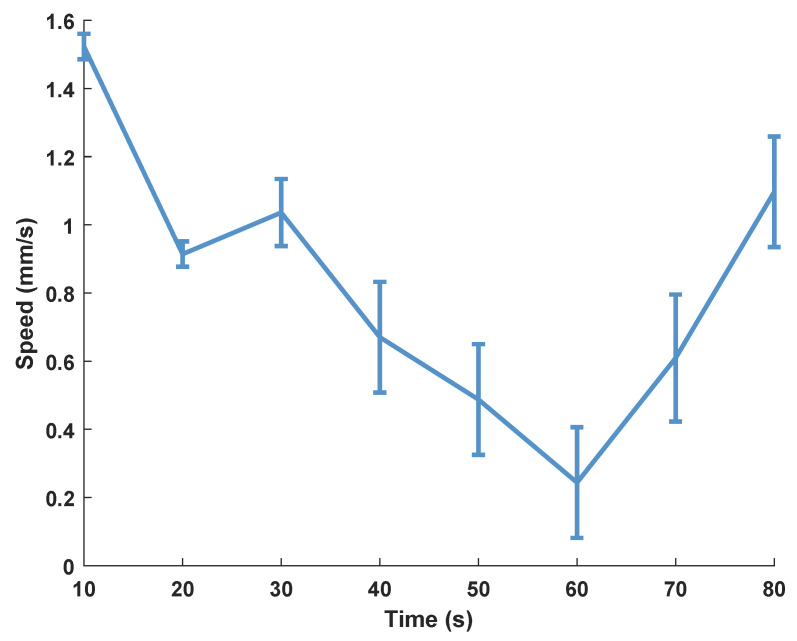
The locomotion speed of the soft octopodal robot on the surface of a pig colon.

Generally, the soft robots exhibit limited movement on biological tissue surfaces due to the relatively high viscosity of the surface. As shown in Figure 8d, the experimental results confirm that the soft robot proposed in this study is capable of locomotion on the surface of a pig colon, which is covered by a layer of mucus. Moreover, to provide a greater actuation force, the robot is driven by a rotating permanent magnet. Results show that the robot achieves an average speed of 0.823 mm/s, as shown in Figure 9.

It is notable that a rotating permanent magnet is used to control the robot’s motion in Figure 5, which leads to a more pronounced body deformation to demonstrate the robot’s flexibility and bending capabilities. Alternatively, in Figure 8, the robot’s drug carrying demonstration is achieved by controlling its motion using an electromagnetic coil with a magnetic field strength of 9 mT. The magnetic field in this case is smaller, inducing less deformation of the robot’s body. Meanwhile, the robot can maintain a more stable and controlled motion while carrying the drug payload.

In addition, the locomotion efficiency and stability of the proposed robot can also be influenced by leg design, in addition to leg count, body mass, the applied magnetic field strength, and its rotation frequency. Indeed, the design of the leg tips can play a crucial role in determining the performance of legged locomotion systems. By optimizing the leg-tip design, it may be possible to improve such aspects as traction, grip, and overall maneuverability. This could potentially result in enhanced locomotion efficiency and stability for our robotic system. The impact of leg-tip design on the robot’s locomotion will be investigated in the future work.

## 4. Conclusions

In this paper, a fast and highly environment-adaptive magnetic soft octopodal robot is designed, which is easy to manufacture with a simple structure. The experimental results verify that the robot can move at a high speed on paper, wood, and PMMA surfaces. It can move freely in an in vitro biomedical environment. This is enabled by its special magnetization, which makes the robot lift its body off the floor while moving. In the meantime, the rotating permanent magnets are controlled to provide a greater driving force, which allows the robot to carry heavier loads. As demonstrated by the experiments, the small, untethered soft robot has great potential for further advancement, especially in the biomedical field, including drug delivery, environmental exploration, and biological sampling. Future work will focus on improving the structural design of the soft robot, enhancing the accuracy of magnetization, and adding a sensory system to the robots for adapting to the environment autonomously.

## Figures and Tables

**Table 1:** Average speed and body lengths (B.L.) covered per second of the octopodal robot during three iterations of motion testing experiments.

Material	Speed (mm/s)	B.L. per Second
Paper	1.135±0.06	0.028
Wood	1.604±0.04	0.040
PMMA	0.536±0.05	0.013

**Table 2:** Comparison of control mechanism and speed of soft robots in the literature.

Robot Type	Control Mechanism ^1^	Surface Material	Relative Speed (Body Length/s)	Reference
Millipede	Magnet	Not specified	1.68	[7]
Inchworm	Magnet(43 mm/s)	Not specified	1.1	[39]
Inchworm	Magnet(18 mm/s)	Acrylic	0.125	[40]
Quadruped	12 mT 0.3 Hz	Wood	0.087	[36]
Millipede	25 mT 0.8 Hz	Wood	0.011	[36]
Hexapod	12 mT 0.5 Hz	Not specified	0.025	[37]
Octopod	5 mT 4.0 Hz	Wood	0.040	This work

^1^ In the context of magnetically controlled robots, the speed of permanent magnet movement is annotated, while the magnitude and frequency controlled by electromagnetic coils are also annotated.

## Data Availability

All data are available in the main text.

## Abbreviations

The following abbreviations are used in this manuscript:

B.L.	Body lengths
DOF	Degree of freedom
MCP	Magnetic polymer composite
PMMA	Polymethyl methacrylate
